# Reliability of Automated Amyloid PET Quantification: Real-World Validation of Commercial Tools Against Centiloid Project Method

**DOI:** 10.3390/tomography11080086

**Published:** 2025-07-30

**Authors:** Yeon-koo Kang, Jae Won Min, Soo Jin Kwon, Seunggyun Ha

**Affiliations:** 1 Division of Nuclear Medicine, Department of Radiology, Seoul St. Mary’s Hospital, College of Medicine, The Catholic University of Korea, Seoul 06591, Republic of Korea; yeonkoo@catholic.ac.kr (Y.-k.K.);; 2 Division of Nuclear Medicine, Department of Radiology, Eunpyeong St. Mary’s Hospital, College of Medicine, The Catholic University of Korea, Seoul 03312, Republic of Korea

**Keywords:** dementia, amyloid PET, Centiloid, artificial intelligence

## Abstract

**Background:** Despite the growing demand for amyloid PET quantification, practical challenges remain. As automated software platforms are increasingly adopted to address these limitations, we evaluated the reliability of commercial tools for Centiloid quantification against the original Centiloid Project method. **Methods:** This retrospective study included 332 amyloid PET scans (165 [^18^F]Florbetaben; 167 [^18^F]Flutemetamol) performed for suspected mild cognitive impairments or dementia, paired with T1-weighted MRI within one year. Centiloid values were calculated using three automated software platforms, BTXBrain, MIMneuro, and SCALE PET, and compared with the original Centiloid method. The agreement was assessed using Pearson’s correlation coefficient, the intraclass correlation coefficient (ICC), a Passing–Bablok regression, and Bland–Altman plots. The concordance with the visual interpretation was evaluated using receiver operating characteristic (ROC) curves. **Results:** BTXBrain (R = 0.993; ICC = 0.986) and SCALE PET (R = 0.992; ICC = 0.991) demonstrated an excellent correlation with the reference, while MIMneuro showed a slightly lower agreement (R = 0.974; ICC = 0.966). BTXBrain exhibited a proportional underestimation (slope = 0.872 [0.860–0.885]), MIMneuro showed a significant overestimation (slope = 1.053 [1.026–1.081]), and SCALE PET demonstrated a minimal bias (slope = 1.014 [0.999–1.029]). The bias pattern was particularly noted for FMM. All platforms maintained their trends for correlations and biases when focusing on subthreshold-to-low-positive ranges (0–50 Centiloid units). However, all platforms showed an excellent agreement with the visual interpretation (areas under ROC curves > 0.996 for all). **Conclusions:** Three automated platforms demonstrated an acceptable reliability for Centiloid quantification, although software-specific biases were observed. These differences did not impair their feasibility in aiding the image interpretation, as supported by the concordance with visual readings. Nevertheless, users should recognize the platform-specific characteristics when applying diagnostic thresholds or interpreting longitudinal changes.

## 1. Introduction

Amyloid PET is recognized as reliable and is currently the only clinically applicable imaging biomarker for detecting the amyloid-β deposition, a pathological hallmark of Alzheimer’s disease (AD) [1,2]. Three radiopharmaceuticals—[^18^F]Florbetaben (FBB), [^18^F]Flutemetamol (FMM), and [^18^F]Florbetapir—are currently available under the U.S. Food and Drug Administration (FDA) approval for amyloid imaging. Although these tracers show broadly similar imaging characteristics and diagnostic performances, they exhibit subtle differences in tracer kinetics and regional distributions [3,4]. Consequently, when using conventional quantitative metrics such as the standardized uptake value ratio (SUVR), tracer- and protocol-specific reference values and thresholds are required. To address this challenge, the Centiloid Project, under the Global Alzheimer’s Association Interactive Network (GAAIN), developed a universal quantitative scale [5]. This system standardizes the whole cerebellum (WC)-referenced SUVR, defining a value of 0 for the average of young cognitively normal individuals and 100 for the average of AD patients, based on 50–70 min [^11^C]PiB PET data. Subsequently, tracer-specific conversion formulas were established to translate PET using ^18^F-labeled radiopharmaceuticals into Centiloid units, providing a cross-tracer standardized quantitative metric [6,7,8,9]. Today, the Centiloid scale is the most widely used quantitative index, offering objective cutoffs, supporting visual assessments, and guiding treatment decisions [10,11,12].

Recently, amyloid-targeting therapies, such as lecanemab and donanemab, have been FDA-approved and have entered clinical use [13,14]. Clinical trials defined eligibility using the amyloid positivity determined by cerebrospinal fluid biomarkers or amyloid PET, and demonstrated significant Centiloid value reductions with treatment, supporting its role in monitoring responses. Particularly, the donanemab trial set a Centiloid threshold of 37 for eligibility and defined treatment discontinuation criteria based on Centiloid value reductions [14,15], indicating its potential pivotal roles in the era of amyloid-targeting drugs.

Despite the increasing clinical demand, the routine application of the Centiloid scale in daily practice faces hurdles, including researcher labor, long analysis times, and the standardization of the analysis process. To address this, multiple automated quantification platforms have been developed and are increasingly used in research and clinical settings. However, the evidence validating their reliability in real-world populations remains limited. Several previous studies have compared amyloid PET quantification software, but most focused on research-use-only tools requiring manual labor, assessed SUVR values without Centiloid standardization, or lacked a reference method (Table 1).

In this study, we investigated the reliability of the Centiloid scale as implemented by three commercial software platforms, each based on distinct processing pipelines currently used in clinical and research practice. We simultaneously applied these software tools to patient amyloid PET scans and compared their Centiloid values with those calculated using the original Centiloid Project method to evaluate their agreement and reliability.

## 2. Materials and Methods

### 2.1. Patients

**Figure 1:**
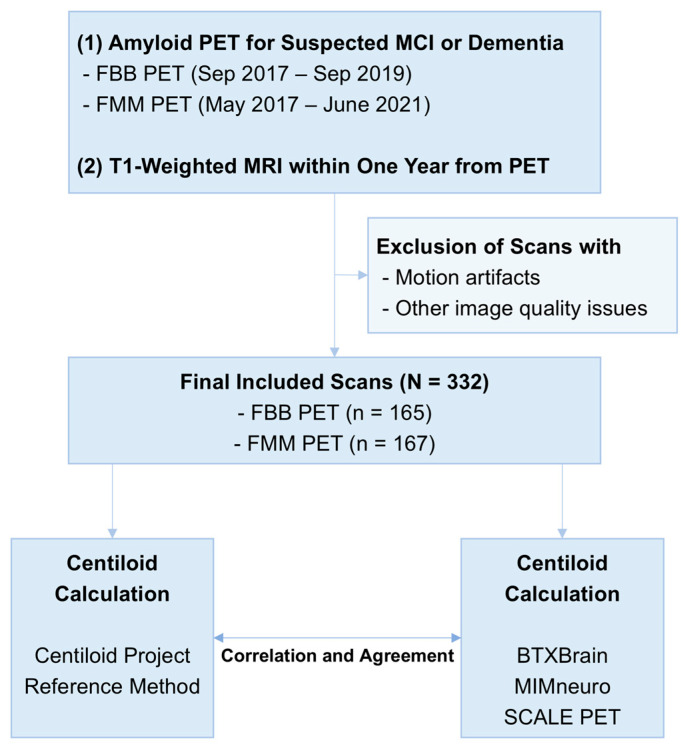
Overall study flow.

This single-center retrospective study included patients who underwent amyloid PET for suspected mild cognitive impairment or dementia. Inclusion criteria were as follows: (1) FBB PET acquired between September 2017 and September 2019 or FMM PET acquired between May 2017 and June 2021 and (2) T1-weighted MRI performed within one year of the PET scan (Figure 1). The inclusion periods were defined separately for each radiotracer to ensure a balanced enrollment of patients across tracers, reflecting differences in their respective introduction dates and usage frequencies in this period. Patients were excluded based on manual review if their PET or MRI images were compromised by motion artifacts or other quality issues that could interfere with image analysis.

### 2.2. Amyloid PET Imaging and Visual Interpretation

Amyloid PET was performed 90 min after intravenous administration of 296 MBq FBB or 185 MBq FMM. PET images were acquired for 20 min using dedicated PET/CT scanners (Discovery 710, GE HealthCare, Chicago, IL, USA; Biograph mCT 40 TruePoint, Siemens Healthineers, Erlangen, Germany) to cover the entire brain immediately following non-contrast CT. PET images were reconstructed using ordered subset expectation maximization (4 iterations and 16 subsets for Discovery; 3 iterations and 21 subsets for Biograph), accompanied by CT-based attenuation correction, scatter correction, and point spread function recovery. Time-of-flight was not applied. The images had a matrix size of 256 × 256.

Visual assessment to determine amyloid positivity was conducted by two experienced nuclear medicine physicians blinded to Centiloid values. Any discrepancies were resolved by thorough consensus discussion.

### 2.3. The Centiloid Quantification Using the Original Centiloid Project Method

Centiloid values were calculated for each amyloid PET image following the standard method established by Centiloid Project (https://www.gaain.org/centiloid-project) (accessed on 16 April 2025). Ref. [5] and used as the reference standards for evaluating the reliability of automated software.

Image processing was performed using SPM8 (Wellcome Trust Centre for Neuroimaging, London, UK; https://www.fil.ion.ucl.ac.uk/spm/software/spm8) (accessed on 16 April 2025) and MATLAB 2024b (Mathworks, Natick, MA, USA). Initially, PET and T1-weighted MRI images were reoriented to approximately match the coordinate space and orientation of the Montreal Neurological Institute (MNI) template. MRI images were coregistered with the MNI template for rough alignment, followed by coregistration of PET images to the corresponding MRI scans. Unified segmentations were applied to the MRI, and PET images were spatially normalized using the transformation parameters derived from segmentation. Mean counts for the cerebral cortex and WC were extracted using the regions of interest (ROIs) provided by Centiloid Project. SUVRs were calculated as the cerebral-to-cerebellar mean count ratio.

The SUVRs were converted to Centiloid values using previously established equations [6,7].

$$Centiloid=\left(153.4\times SUVR\right)-154.9,\;\mathrm{for}\;\mathrm{FBB}$$

$$Centiloid=\left(121.42\times SUVR\right)-121.16,\;\mathrm{for}\;\mathrm{FMM}$$

### 2.4. Automated Software-Based Centiloid Quantification

Centiloid values were also calculated using three commercially available software platforms: BTXBrain (v1.1.2, Brightonix Imaging, Seoul, Republic of Korea), MIMneuro (v.7.3.7, MIM Software, Cleveland, OH, USA), and SCALE PET (v2.0.1, Neurophet, Seoul, Republic of Korea). All DICOM images were imported into the software platforms and processed to calculate Centiloid values without any preprocessing, including partial-volume correction or harmonization of image quality across vendors.

Briefly, BTXBrain performs fully automated PET-only processing, using an in-house artificial intelligence (AI)-based spatial normalization pipeline without requiring MRI [23]. It applies the original Centiloid Project ROIs to calculate global SUVR with WC reference and converts SUVRs to Centiloid values using the original equations. In addition to the official BTXBrain Centiloid values, we provided a recalibrated Centiloid value adapted using GAAIN dataset as Supplementary Materials, which was a local setting in our institution.

MIMneuro also operates as a PET-only platform, registering PET images via BrainAlign deformable registration algorithm with multiple template registration of negative, average, and positive amyloid-specific templates [17]. The global cortical SUVR was calculated using to the [^18^F]Florbetapir Clark atlas referenced to WC [24]. The extracted SUVRs were converted to Centiloid values using recalibrated equations tailored to its pipeline [8].

SCALE PET offers PET-only and MRI-based versions, and the MRI-based version was used in this study. This version uses AI-based segmentation for patient MRI images directly on the individual space without transforming the MRI to a template [25]. The PET image is registered to the individual 3D T1 MRI using affine registration, and global SUVRs with WC reference are calculated on individualized ROIs based on the Desikan–Killiany atlas [21]. Centiloid values are calculated using an equation recalibrated with public datasets [5,6,7,8,26].

### 2.5. Statistical Analysis

Patient characteristics were summarized as mean ± standard deviation for continuous variables and as numbers with percentages for categorical variables. Correlations between reference and software-derived Centiloid values were evaluated using Pearson’s correlation coefficients. The reliability of each tool was assessed using intraclass correlation coefficient (ICC), and agreement was visualized using Bland–Altman plots. Passing–Bablok regression was performed to assess systematic and proportional bias. Differences between Pearson’s correlation coefficient were tested using Fisher’s Z-transformation. Concordance with visual interpretation results was analyzed using receiver operating characteristic (ROC) curve analysis, and optimal cutoff values were determined based on Youden’s index. A *p*-value < 0.05 was considered significant.

## 3. Results

### 3.1. Patient Characteristics

This study included 332 patients (age, 73.9 ± 7.5 years; male/female = 107:225). A total of 165 patients underwent FBB PET, and 167 patients underwent FMM PET (Table 2). The age and sex distribution were not significantly different between the two tracer groups. The Clinical Dementia Rating–Sum of Boxes (CDR-SB) showed no significant difference between groups (3.87 ± 3.13 vs. 3.86 ± 3.72, *p* = 0.983). The proportion of amyloid-positive cases based on the visual reading was also similar (44.2% for FBB and 41.9% for FMM, *p* = 0.751). The mean Centiloid value based on the original method was 35.9 ± 49.2 for FBB and 28.8 ± 38.4 for FMM, and the difference was not significant (*p* = 0.146).

### 3.2. The Agreement of Software-Based Centiloid with the Reference

**Figure 2:**
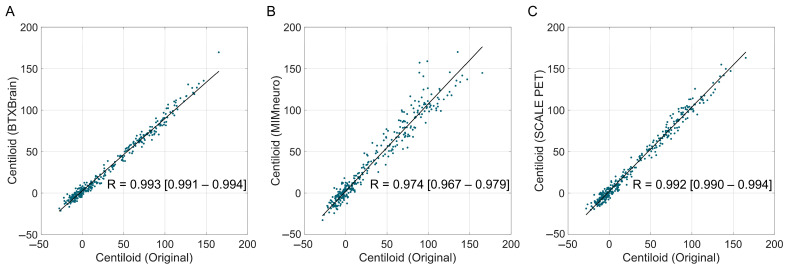
Correlation between software-based and original Centiloid values. Scatter plots showing the correlation between the reference Centiloid values calculated using the original Centiloid Project method and Centiloid values derived from three commercial software platforms ((**A**), BTXBrain; (**B**), MIMneuro; (**C**), SCALE PET). Each dot represents an individual patient scan. The solid lines represent the Passing–Bablok regression lines.

Overall, software-derived Centiloid values demonstrated a reliable correlation with the reference (Figure 2). BTXBrain and SCALE PET achieved an excellent linear association (R = 0.993 [95% CI, 0.991–0.994] and 0.992 [0.990–0.994], respectively), whereas MIMneuro showed a slightly lower correlation (R = 0.974 [0.967–0.979]) with a significant difference with the other two platforms (*p* < 0.001 for both) (Table 3). The Passing–Bablok regression showed a proportional bias in each platform: BTXBrain demonstrated a proportional underestimation (slope = 0.872 [0.860–0.885]), SCALE PET exhibited near-unity scaling with an equivocal overestimation (slope = 1.014 [0.999–1.029]), and MIMneuro showed a proportional overestimation (slope = 1.053 [1.026–1.081]). In terms of the overall reliability presented as ICCs, SCALE PET achieved the highest ICC (0.991 [0.983–0.994]), followed by BTXBrain (0.986 [0.982–0.989]) and MIMneuro (0.966 [0.944–0.978]).

**Figure 3:**
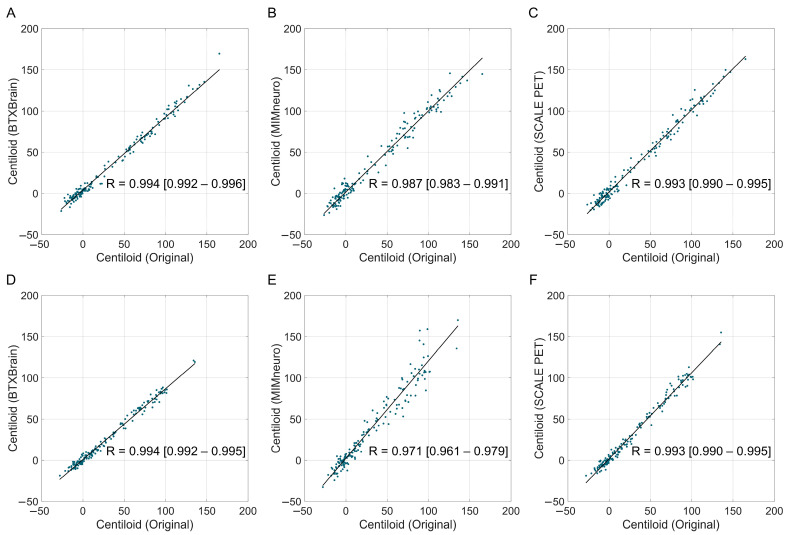
Correlation between software-based and original Centiloid values by radiotracer. The scatter plots show the correlation between the reference Centiloid values calculated using the original Centiloid Project method and Centiloid values derived from three commercial software platforms ((**A**,**D**): BTXBrain; (**B**,**E**): MIMneuro, (**C**,**F**): SCALE PET), separated by radiotracers ((**A**–**C**): [^18^F]Florbetaben PET; (**D**–**F**): [^18^F]Flutemetamol PET). Each dot represents an individual patient scan. The solid lines represent the Passing–Bablok regression lines.

In tracer-specific analyses, both BTXBrain and SCALE PET maintained an excellent performance for FBB (BTXBrain, R = 0.994, ICC = 0.989; SCALE PET, R = 0.993, ICC = 0.992) and FMM (BTXBrain, R = 0.994, ICC = 0.980; SCALE PET, R = 0.993, ICC = 0.988) (Figure 3 and Table 3). MIMneuro’s agreement was robust for FBB (R = 0.987, ICC = 0.986) but declined for FMM (R = 0.971, ICC = 0.941). For the FMM subgroup, both SCALE PET and MIMneuro overestimated Centiloid values; however, MIMneuro’s overestimation (slope = 1.182 [1.130–1.231]) was greater than that of SCALE PET (slope = 1.044 [1.022–1.067]). In contrast, BTXBrain consistently underestimated Centiloid values across tracers, although the underestimation was slightly more pronounced in FMM (FBB, slope = 0.883 [0.867–0.899]; FMM, slope = 0.859 [0.842–0.877]).

Using 10–30 Centiloids as a gray zone, we assessed the presence of critically discordant cases, defined as cases where Centiloid values were classified as definitely negative (<10) by the original method but definitely positive (>30) by the software or vice versa. Despite the quantitative biases and variability, none of the tools produced such a discrepancy.

### 3.3. The Agreement of Software-Based Centiloid with the Original Method According to Centiloid Levels

**Figure 4:**
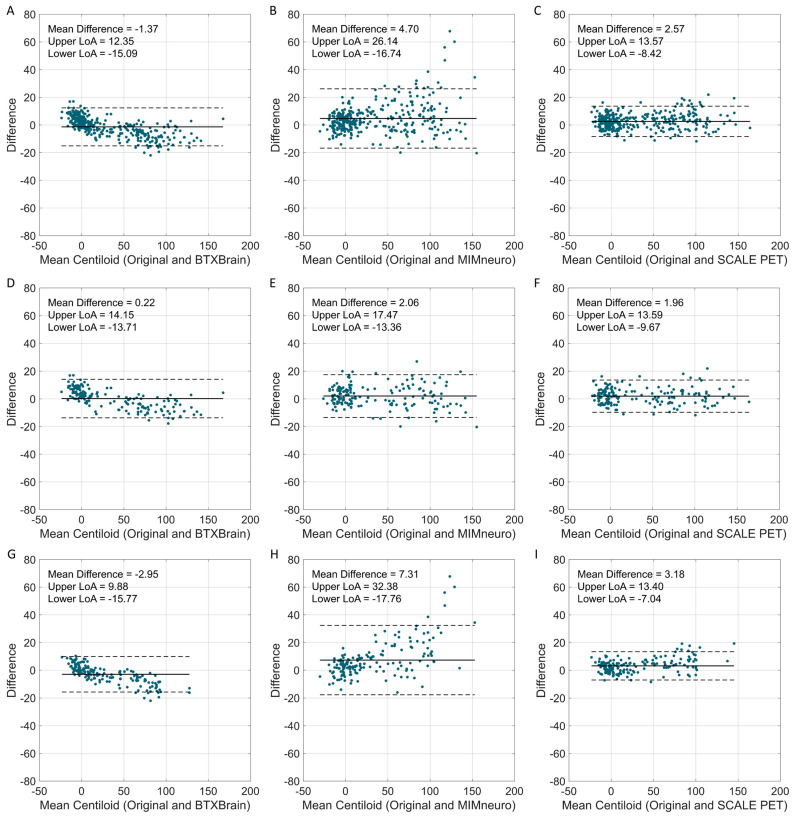
Bland–Altman plots comparing software-derived Centiloid values with the original Centiloid values. Bland–Altman plots show the agreement between software-derived Centiloid values and the original Centiloid Project method. (**A**–**C**) Total population (BTXBrain, MIMneuro, SCALE PET); (**D**–**F**) [^18^F]Florbetaben (BTXBrain, MIMneuro, SCALE PET); (**G**–**I**) [^18^F]Flutemetamol (BTXBrain, MIMneuro, SCALE PET). Each plot displays the mean Centiloid value on the x-axis and the difference between software-derived and reference Centiloid values on the y-axis. The solid line represents the mean difference, while the dashed lines indicate the 95% limits of agreement (LoAs, mean difference ± 1.96 standard deviations).

Beyond the overall agreement, we further examined biases across Centiloid ranges. Bland–Altman plots across the full Centiloid spectrum revealed that limits of agreement (LoAs) were narrowest for SCALE PET (−8.4 to 13.6), followed by BTXBrain (−15.1 to 12.4) and MIMneuro (−16.7 to 26.1) (Figure 4). BTXBrain exhibited a bidirectional bias, showing a slight overestimation at low Centiloid values and a progressive underestimation at higher values, which is consistent with its lower slope in the regression. MIMneuro showed a positive bias across all ranges, with larger differences in the definitely positive range. SCALE PET demonstrated a borderline overestimation over the entire range.

**Figure 5:**
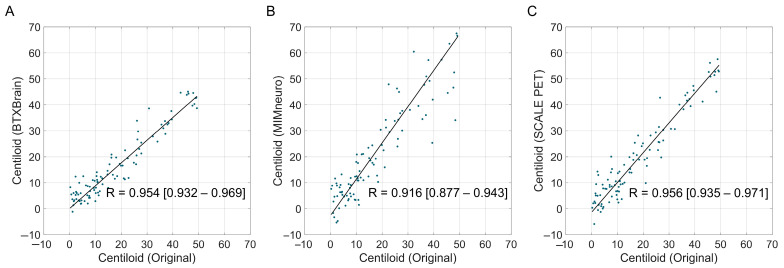
The correlation between software-derived and original Centiloid values in the subthreshold-to-low-positive range (0–50 Centiloid unit). Scatter plots show the correlation between the reference Centiloid values calculated using the original Centiloid Project method and Centiloid values derived from three commercial software platforms ((**A**), BTXBrain; (**B**), MIMneuro; (**C**), SCALE PET), limited to cases with original Centiloid values between 0 and 50. Each dot represents an individual patient scan. The solid lines represent the Passing–Bablok re-gression lines.

We focused on the subthreshold-to-low-positive range (0–50 Centiloid unit), which is extended from the commonly considered gray zone (typically 10–30) [11], to evaluate the reliability in the most clinically significant zone for aiding diagnosis. Similar patterns were observed in this population, although overall statistics for reliability were slightly decreased due to the smaller group size (Figure 5 and Table 4). BTXBrain showed a strong agreement (R = 0.954 [0.932–0.969]; ICC = 0.942 [0.906–0.964]) with a modest underestimation (mean difference = −1.6 [−2.5 to −0.7]; slope = 0.876 [0.817–0.930]). SCALE PET demonstrated excellent reliability (R = 0.956 [0.935–0.971]; ICC = 0.944 [0.911–0.964]), with a modest overestimation (mean difference = +1.6 [0.6–2.6]; slope = 1.147 [1.078–1.210]). MIMneuro’s performance was relatively lower (R = 0.916 [0.877–0.943]; ICC = 0.853 [0.740–0.912]), with a more pronounced overestimation (mean difference = +4.0 [2.3–5.7]; slope = 1.397 [1.261–1.507]).

When stratified by tracers, MIMneuro’s overestimation was accentuated in the FMM subgroup (slope = 1.436 [1.318–1.584]; mean difference = +5.9 [3.6–8.1]). SCALE PET’s slight overestimation was consistent across tracers (FBB slope = 1.155 [0.978–1.355]; FMM slope = 1.146 [1.074–1.215]; mean difference = +1.6 [0.6–2.7] for both), although statistical significance was achieved only in FMM due to the group size difference. BTXBrain tended to underestimate Centiloid values for both tracers, with the underestimation more prominent in FMM (slope = 0.890 [0.826–0.941]) compared to FBB (slope = 0.922 [0.776–1.093]).

### 3.4. Agreement with Visual Interpretation Results

**Figure 6:**
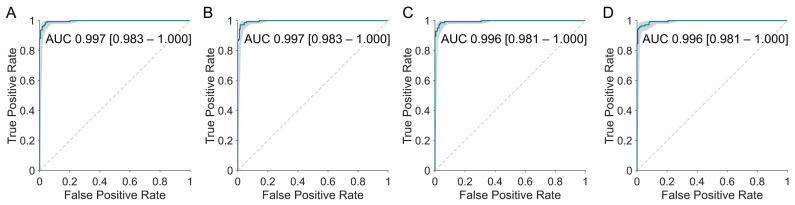
Receiver operating characteristic (ROC) curves for predicting amyloid PET visual interpretation results using Centiloid values derived from each software platform. The original method (**A**) and all three platforms—BTXBrain (**B**), MIMneuro (**C**), and SCALE PET (**D**)—demonstrated an excellent concordance with the visual interpretation, with areas under the curve (AUCs) exceeding 0.996.

All software-derived Centiloids demonstrated an excellent concordance with the visual interpretation, comparable to the original method (Table 5 and Figure 6). The area under the ROC curve (AUROC) was high (Original 0.997 [95% CI, 0.983–1.000]; BTXBrain 0.997 [0.983–1.000]; MIMneuro 0.996 [0.981–1.000]; SCALE PET 0.996 [0.981–1.000]), even for MIMneuro which exhibited a relatively lower correlation with the original method.

Optimal cutoffs determined by Youden’s index were close overall to the original threshold of 21.4. BTXBrain’s cutoff (21.3) was the most similar to the reference. MIMneuro (28.2) and SCALE PET (25.4) required higher cutoff values.

When defining a practical diagnostic threshold and gray zone—i.e., cutoffs that achieve either 99% sensitivity or 99% specificity—the ranges for SCALE PET (18.8 for 99% sensitivity and 30.8 for 99% specificity) closely mirrored those of the original method (19.9–30.8). BTXBrain (16.6–37.6) and MIMneuro (21.1–38.1), however, needed substantially wider ranges to attain an equivalent sensitivity- or specificity-driven performance.

## 4. Discussion

Since its introduction in 2015 [5], the Centiloid framework has become the most widely accepted tracer-independent quantitative metric for amyloid PET, supporting amyloid positivity determination [27,28], disease progression prediction [29], amyloid burden increase monitoring [30], therapy candidate selection [14,15], treatment response evaluation [13,14,31,32], and treatment discontinuation decisions [14]. With the shift from clinical to biomarker-based diagnostic criteria for AD and the rise of amyloid-targeting therapies, the demand for practical quantification methods has grown, prompting the introduction of several commercial tools for clinical use [33,34,35]. Although commercial software adopting the Centiloid scale has demonstrated a satisfactory performance on reference datasets before market release, the validation using real-world data remains limited. While prior studies compared software-generated Centiloid values across platforms [19,22], none have benchmarked multiple commercial tools directly against the original reference method.

We addressed this gap by evaluating the reliability of Centiloid values generated by three widely used commercial tools, using the original Centiloid Project method as the reference standard. All three platforms produced Centiloid values with an acceptable agreement, but software-specific differences were evident. BTXBrain demonstrated an excellent overall correlation with the reference but exhibited a proportional underestimation. MIMneuro showed a slightly lower correlation and greater random variability, with a particularly pronounced overestimation in FMM scans. SCALE PET achieved an excellent overall correlation and minimal proportional overestimations for FMM. Importantly, these differences did not compromise the concordance with visual interpretations, suggesting that a strict alignment with the original method may not be necessary for maintaining clinical diagnostic performances.

Among the three platforms, SCALE PET showed the strongest concordance with the original Centiloid method, with an excellent correlation and a minimal proportional bias (Table 6). Although a slight overestimation was observed (slope 1.044 in FMM; mean difference +1.6), this deviation is clinically negligible. This excellent reliability reflects both the consistency of the AI-based individual MRI segmentation used for the PET SUVR quantification and its appropriate recalibration to match the original Centiloid. This robustness makes SCALE PET an attractive option for obtaining Centiloid values that most closely align with the reference. The MRI-based AI protocol for PET quantification ensures dependable results with a modest 5–10 min processing time, but it relies on having a separately acquired MRI. It is noteworthy that SCALE PET also offers a PET-only version [21], which warrants further validation to assess its performance in Centiloid calculations without MRI input.

BTXBrain demonstrated an excellent correlation with the Centiloid reference, though it initially underestimated values, mainly in the positive amyloid PET range, because it applies the original Centiloid equation without adjustments. In practice, this underestimation stems from a SUVR mismatch between BTXBrain and the MRI-based SPM pipeline [36], whereas BTXBrain’s SUVR aligns more closely with FreeSurfer, and both BTXBrain and FreeSurfer outperform SPM in spatial registration accuracy [23]. After applying a linear recalibration formula based on the GAAIN dataset to align with the original Centiloid scale, as MIMneuro and SCALE PET do, BTXBrain proves efficient for both clinical and research applications, with supplementary correlation plots (Figure S1), Bland–Altman analyses (Figure S2), and a post-recalibration concordance with visual reads (Table S1). Its ability to generate robust, highly correlated Centiloid results in about one to three minutes—without requiring a patient MRI—constitutes a clear practical advantage.

MIMneuro exhibited a higher variability and the lowest correlation with the original Centiloid method among the evaluated pipelines. Its PET quantification relies on an adaptive-template registration with a landmark-based deformation algorithm, which can be less precise than MRI-guided methods, such as the MRI-based high-dimensional normalization (SPM), MRI-driven surface registration and cortical parcellation (FreeSurfer), MRI-driven deep-learning-based tissue segmentation for ROI extractions (SCALE PET), or MR-less end-to-end PET normalization via deep learning trained on paired PET-MRI data (BTXBrain). Importantly, despite this variability, MIMneuro maintained a concordance with the visual interpretation, indicating that these methodological differences do not reduce its essential clinical diagnostic value. However, in research or clinical trial settings, where Centiloid values must be rigorously compared and reproduced, its higher variability and lower correlation with the original Centiloid method relative to other software should be taken into account.

Various Centiloid thresholds have been proposed for determining amyloid positivity [15,28,37,38], but given the known variability, applying a single cutoff across all settings is generally not recommended [11]. In this study, we suggested platform-specific cutoffs corresponding to a 99% sensitivity and a 99% specificity, which aligned broadly with the published threshold, which ranges from 17 to 42. This supports the overall suitability of these commercial tools for clinical and research use. Nevertheless, the small but consistent differences in cutoffs across platforms indicate the need for caution when applying a fixed Centiloid threshold (e.g., 30 for treatment enrollment decisions) across the different software. A careful consideration of platform-specific characteristics is essential for accurate and clinically meaningful interpretations.

We excluded comparisons of the SUVR between software platforms due to differences in their atlases. BTXBrain uses the Centiloid Project atlas for calculating Centiloid values, but it applies the Automated Anatomical Labeling 3 atlas for SUVR values displayed within the software. MIMneuro employs an atlas developed for the [^18^F]Florbetapir PET quantification [24], and SCALE PET uses the Desikan–Killiany atlas commonly adopted in MRI segmentation [39]. These differences in the ROI parcellation introduce an unavoidable SUVR variability; therefore, we focused our analysis exclusively on Centiloid values.

This study has several limitations. First, the single-center retrospective design limits the exclusion of a potential selection bias. To ensure a broader generalizability and robustness, future multicenter studies are warranted to validate these automated Centiloid platforms across diverse populations and imaging systems.

The analysis did not include images acquired from digital PET scanners, which are increasingly adopted in clinical practice. Because digital PET systems differ in technical aspects, such as the spatial resolution, sensitivity, and noise characteristics, the performance of automated Centiloid quantification tools may vary accordingly. As a result, the generalizability of our findings to digital PET systems remains uncertain.

In addition, our study focused on validating the quantitative outputs of each software platform, rather than examining their associations with clinical outcomes. As such, the comparative clinical utility of these tools, such as predicting cognitive decline or monitoring treatment responses, remains outside the scope of this analysis. This design reflects our primary aim: to evaluate whether Centiloid values derived from recently commercialized, automated platforms can be used interchangeably with those obtained from the reference method and other established approaches. Confirming their technical reliability is a necessary prerequisite before applying these Centiloid values for outcome prediction or treatment monitoring.

Last, we evaluated the commercial software in a clinical setting without harmonizing PET/CT images across different scanners. Despite using PET/CT systems from multiple vendors, the results were highly consistent, suggesting that Centiloid values from these commercial platforms can be used reliably without prior harmonization.

## 5. Conclusions

This study is the first to directly benchmark dedicated, nearly fully automated software platforms for Centiloid calculations against the original Centiloid Project method using real-world amyloid PET data. By evaluating tools with distinct processing pipelines, it provides a practical reference for researchers and clinicians to assess the reliability of Centiloid values generated by different platforms. All three commercial automated platforms provided an acceptable reliability for the Centiloid quantification when compared to the original Centiloid Project method. Although each software exhibited a proportional bias or variability, these differences did not compromise the agreement with the visual interpretation, suggesting their potential utility as supportive tools in clinical practice. Nevertheless, clinicians should remain aware of platform-specific characteristics, especially when applying diagnostic thresholds or monitoring longitudinal changes. Further investigations are warranted, including large-scale multicenter validation studies that incorporate digital PET/CT systems and assess associations between software-derived Centiloid values and clinical outcomes, to establish their utility in patient management.

## Figures and Tables

**Table 1:** Previous studies comparing amyloid PET quantification software.

Author (Year)	Platforms	Metrics	Key Findings	Limitations	Ref.
C Hutton et al. (2015)	SPM; Syngo.via	SUVR	Good overall agreement	No Centiloid calculation; single new platform	[16]
WH Choi et al. (2016)	PMOD; MIMneuro	SUVR	Good overall agreement; regional difference	No Centiloid calculation; Single new platform	[17]
MR Battle et al. (2018)	SPM; PMOD; FSL	SUVR; Centiloid	Centiloid conversion equation according to each platform	Confined to conventional research platforms	[6]
SM Landau et al. (2022)	FreeSurfer; SPM	SUVR	Good overall agreement between MRI-dependent and MRI-free processing	Confined to conventional research platforms; no Centiloid calculation	[18]
A Jovalekic et al. (2023)	15 platforms (6 for Centiloid)	SUVR; Centiloid	Descriptive analysis of values from each platform	No evaluation of correlation or inter-platform agreement	[19]
HW Roh et al. (2023)	PMOD; Heuron	SUVR	Good overall agreement	No Centiloid calculation; single new platform	[20]
S Kim et al. (2024)	Syngo.via; SCALE PET	SUVR	High predictive value for visual interpretation results in both platforms	No Centiloid calculation; no comparison with reference	[21]
C Shang et al. (2024)	CapAIBL; VIZCalc; Amyquant	Centiloid	Strong correlations among values using the three methods	No comparison with reference	[22]

Abbreviations: Ref, reference; SUVR, standardized uptake value ratio.

**Table 2:** Patient characteristics.

Factor	Florbetaben (n = 165)	Flutemetamol (n = 167)	Total (n = 332)	t/χ^2^	*p*
Age (year)	74.0 ± 7.8	73.8 ± 7.2	73.9 ± 7.5	−0.247	0.805
Sex (male/female)	59:106	48:119	107:225	1.563	0.211
CDR-SB	3.86 ± 3.72	3.87 ± 3.13	3.87 ± 3.44	0.021	0.983
Positive amyloid scan *	73 (44.2%)	70 (41.9%)	143 (47.0%)	0.101	0.751
Centiloid values	35.9 ± 49.2	28.8 ± 38.4	32.3 ± 44.2	−1.456	0.146

Abbreviations: CDR-SB, Clinical Dementia Rating–Sum of Boxes. * Positive amyloid scan was determined by visual reading by two nuclear medicine physicians.

**Table 3:** Agreement of software-based Centiloids according to tracer types.

Method	Slope	Intercept	R	ICC	*p* *
Total (n = 332)					
BTXBrain	0.872 [0.860–0.885]	2.702 [2.308–2.967]	0.993 [0.991–0.994]	0.986 [0.982–0.989]	<0.001
MIMneuro	1.053 [1.026–1.081]	2.297 [1.904–2.778]	0.974 [0.967–0.979]	0.966 [0.944–0.978]	<0.001
SCALE PET	1.014 [0.999–1.029]	2.288 [1.659–2.571]	0.992 [0.990–0.994]	0.991 [0.983–0.994]	<0.001
Florbetaben (n = 165)					
BTXBrain	0.883 [0.867–0.899]	4.195 [3.563–4.859]	0.994 [0.992–0.996]	0.989 [0.984–0.992]	<0.001
MIMneuro	0.984 [0.959–1.009]	1.731 [0.920–2.243]	0.987 [0.983–0.991]	0.986 [0.980–0.990]	<0.001
SCALE PET	0.996 [0.976–1.015]	1.870 [1.457–2.501]	0.993 [0.990–0.995]	0.992 [0.988–0.995]	<0.001
Flutemetamol (n = 167)					
BTXBrain	0.859 [0.842–0.877]	0.932 [0.667–1.345]	0.994 [0.992–0.995]	0.980 [0.964–0.988]	<0.001
MIMneuro	1.182 [1.130–1.231]	2.531 [1.770–2.550]	0.971 [0.961–0.979]	0.941 [0.868–0.968]	<0.001
SCALE PET	1.044 [1.022–1.067]	1.893 [1.012–2.595]	0.993 [0.990–0.995]	0.988 [0.969–0.944]	<0.001

Abbreviations: R, Pearson’s correlation coefficient; ICC, intraclass correlation coefficient. Slope and intercept are based on Passing–Bablok regression analysis. Intraclass correlation coefficient was based on fixed rater effect, absolute agreement, and single measurement. Values in square brackets represent 95% confidence intervals. * *p*-values for correlation coefficients adjusted by Bonferroni correction.

**Table 4:** The agreement of software-based Centiloid values in the subthreshold-to-low-positive range (0–50 Centiloid unit).

Method	Slope	Intercept	R	ICC	Mean Difference	*p* *
Total (n = 98)						
BTXBrain	0.876 [0.817–0.930]	0.111 [−0.630–1.508]	0.954 [0.932–0.969]	0.942 [0.906–0.964]	−1.6 [−2.5–−0.7]	<0.001
MIMneuro	1.397 [1.261–1.507]	−2.562 [−4.803–−0.328]	0.916 [0.877–0.943]	0.853 [0.740–0.912]	4.0 [2.3–5.7]	<0.001
SCALE PET	1.147 [1.078–1.210]	−1.339 [−2.482–0.397]	0.956 [0.935–0.971]	0.944 [0.911–0.964]	1.6 [0.6–2.6]	<0.001
Florbetaben (n = 35)						
BTXBrain	0.922 [0.776–1.093]	2.375 [0.199–3.498]	0.935 [0.874–0.967]	0.933 [0.871–0.965]	0.5 [−1.3–2.2]	<0.001
MIMneuro	1.104 [0.877–1.390]	−1.105 [−4.450–1.808]	0.886 [0.785–0.942]	0.887 [0.788–0.941]	0.6 [−1.8–3.0]	<0.001
SCALE PET	1.155 [0.978–1.355]	−0.219 [−3.112–1.930]	0.921 [0.849–0.960]	0.912 [0.833–0.955]	1.6 [−0.5–3.7]	<0.001
Flutemetamol (n = 63)						
BTXBrain	0.890 [0.826–0.941]	−0.843 [−1.765–1.016]	0.971 [0.953–0.983]	0.948 [0.812–0.978]	−2.7 [−3.6–−1.8]	<0.001
MIMneuro	1.436 [1.318–1.584]	−2.078 [−5.554–−0.128]	0.941 [0.904–0.964]	0.838 [0.597–0.922]	5.9 [3.6–8.1]	<0.001
SCALE PET	1.146 [1.074–1.215]	−1.684 [−2.917–0.567]	0.973 [0.956–0.984]	0.959 [0.926–0.977]	1.6 [0.6–2.7]	<0.001

Abbreviations: R, Pearson’s correlation coefficient; ICC, intraclass correlation coefficient. The intraclass correlation coefficient was based on the fixed rater effect, absolute agreement, and single measurement. The mean difference is defined as (software-derived Centiloid values) − (Centiloid values from the original Centiloid Project method). Values in the square brackets represent 95% confidence intervals. Groups were categorized according to Centiloid values calculated using the original Centiloid Project method. * *p*-values for correlation coefficients adjusted by Bonferroni correction.

**Table 5:** Agreement of Centiloid values derived from variable methods with visual interpretation results.

Method	AUROC	Optimal Cutoff	Sensitivity	Specificity	Cutoff for 99% Sensitivity	Cutoff for 99% Specificity
Original	0.997 [0.983–1.000]	21.4	0.986 [0.950–0.998]	0.963 [0.925–0.985]	19.9	30.8
BTXBrain	0.997 [0.983–1.000]	21.3	0.972 [0.930–0.992]	0.984 [0.954–0.997]	16.6	37.6
MIMneuro	0.996 [0.981–1.000]	28.2	0.986 [0.950–0.998]	0.963 [0.925–0.985]	21.1	38.1
SCALE PET	0.996 [0.981–1.000]	25.4	0.958 [0.911–0.984]	0.984 [0.954–0.997]	18.8	30.8

Abbreviations: AUROC, area under receiver operating characteristics curve. Optimal cutoff values were determined using the Youden index. Values in the square brackets represent 95% confidence intervals.

**Table 6:** A summary of key findings in this study.

Method	Agreement with Reference	Proportional Bias	Concordance with Visual Reading
BTXBrain	R = 0.993, ICC = 0.986 for all; R= 0.994, ICC = 0.989 for FBB; R = 0.994, ICC = 0.980 for FMM	Slope = 0.872 for all; Slope = 0.883 for FBB; Slope = 0.859 for FMM.	AUROC 0.997
MIMneuro	R = 0.974, ICC = 0.966 for all; R = 0.987, ICC = 0.986 for FBB; R = 0.971, ICC = 0.941 for FBB	Slope = 1.053 for all; Slope = 0.984 for FBB; Slope = 1.182 for FMM	AUROC 0.996
SCALE PET	R = 0.992, ICC = 0.991 for all; R = 0.993, ICC = 0.992 for FBB; R = 0.993, ICC = 0.988 for FMM	Slope = 1.014 for all; Slope = 0.996 for FBB; Slope = 1.044 for FMM	AUROC 0.996

Abbreviations: ICC, intraclass correlation coefficient; AUROC, area under the receiver operating characteristic curve; FBB, [^18^F]Florbetaben; and FMM, [^18^F]Flutemetamol.

## Data Availability

The data presented in this study are available on request from the corresponding author. Permission from our institution’s Data Review Board is required prior to sharing.

## Supplementary Materials

The following supporting information can be downloaded at https://www.mdpi.com/article/10.3390/tomography11080086/s1, Figure S1: Correlation plots showing the recalibrated Centiloid values derived from BTXBrain against the original Centiloid Project reference values; Figure S2: Bland–Altman plots comparing the recalibrated Centiloid values derived from BTXBrain with the original Centiloid values; Table S1: Agreement of recalibrated Centiloid values derived from BTXBrain with visual interpretation results.

## Abbreviations

The following abbreviations are used in this manuscript:

AD	Alzheimer’s disease
FBB	[^18^F]Florbetaben
FMM	[^18^F]Flutemetamol
FDA	Food and Drug Administration
SUVR	Standardized uptake value ratio
GAAIN	Global Alzheimer’s Association Interactive Network
WC	Whole cerebellum
MNI	Montreal Neurological Institute
AI	Artificial intelligence
ICC	Intraclass correlation coefficient
ROC	Receiver operating characteristic
CDR-SB	Clinical Dementia Rating–Sum of Boxes
LoAs	Limits of agreement
AUROC	Area under the ROC curve
