# Supplementary material for: Reliability of Automated Amyloid PET Quantification: Real-World Validation of Commercial Tools Against Centiloid Project Method
Source: Tomography. 2025 Jul 30;11(8):86. doi: 10.3390/tomography11080086 (PMC12389775; doi:10.3390/tomography11080086)
Supplement: Supplementary file 1 [file tomography-11-00086-s001.zip › tomography-3732161-supplementary.pdf]

Supplementary Data

The supplementary data present additional analyses evaluating the performance of BTXBrain following recalibration of its SUVR-to-Centiloid conversion equation. The recalibration was performed using the Global Alzheimer's Association Interactive Network (GAAIN) Centiloid Project datasets separately for [^{18}F]Florbetaben and [^{18}F]Flutemetamol.

Recalibrated Centiloid values demonstrated improved agreement with the reference standard and reduced proportional underestimation across total population and each tracer group, as shown in scatter plots (**Figure S1**) and Bland-Altman plots (**Figure S2**).

Table S1 presents concordance with visual interpretation for BTXBrain after recalibration. As recalibration involved a linear transformation of values, the area under the receiver operating characteristic curve (AUROC = 0.997 [0.983 – 1.000]) and the sensitivity and specificity for the optimal cutoff remained unchanged. In addition, the thresholds corresponding to 99% sensitivity and 99% specificity were updated following recalibration, with revised values of 13.7 (99% sensitivity) and 38.2 (99% specificity).

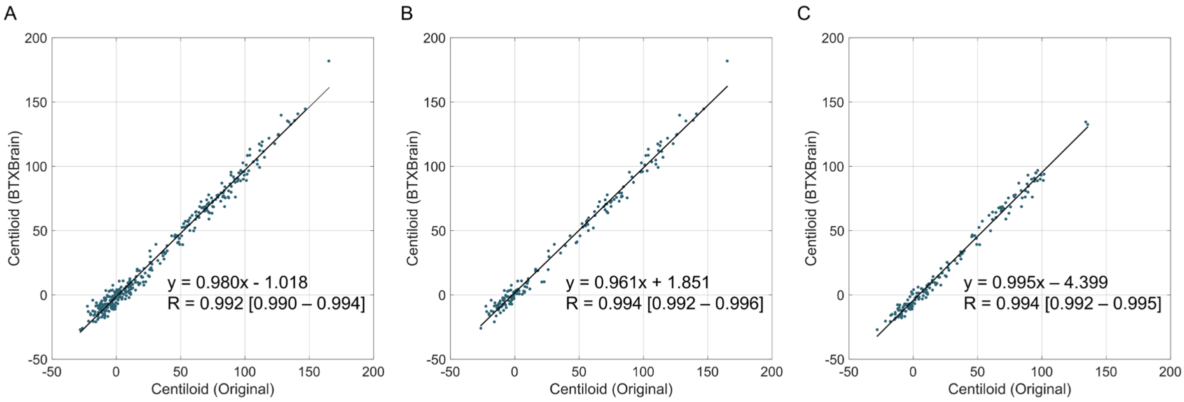

Figure S1. Correlation plots showing the recalibrated Centiloid values derived from BTXBrain against the original Centiloid Project reference values.

The Centiloid calculation equation in BTXBrain was recalibrated using GAAIN Centiloid Project data, and Centiloid values for subjects in this study were recalculated using the updated equation and compared with the standard reference values. Recalibration improved agreement by addressing the proportional underestimation observed in the original BTXBrain outputs for (**A**) Total population, (**B**) [^{18}F]Florbetaben, and (**C**) [^{18}F]Flutemetamol subgroups.

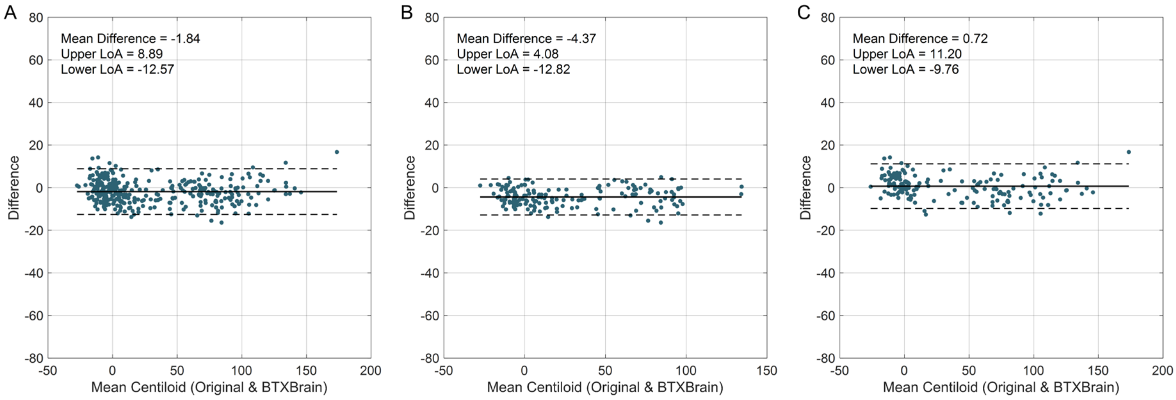

Figure S2. Bland–Altman plots comparing the recalibrated Centiloid values derived from BTXBrain with the original Centiloid values.

Bland–Altman plots showing the agreement between software-derived Centiloid values and the original Centiloid Project method. (**A**) Total population; (**B**) [^{18}F]Florbetaben; (**C**) [^{18}F]Flutemetamol. Each plot displays the mean Centiloid value on the x-axis and the difference between software-derived and reference Centiloid values on the y-axis. The solid line represents the mean difference, while the dashed lines indicate the 95% limits of agreement (LoA, mean difference ± 1.96 standard deviations).

Table S1. Agreement of recalibrated Centiloid values derived from BTXBrain with visual interpretation results

Method	AUROC	Optimal Cutoff	Sensitivity	Specificity	Cutoff for 99% sensitivity	Cutoff for 99% specificity
**Reference**	0.997 [0.983 – 1.000]	21.4	0.986 [0.950 – 0.998]	0.963 [0.925 – 0.985]	19.9	30.8
**BTXBrain**	0.997 [0.983 – 1.000]	21.3	0.972 [0.930 – 0.992]	0.984 [0.954 – 0.997]	16.6	37.6
**BTXBrain_ Recalibrated**	0.997 [0.983 – 1.000]	20.1	0.972 [0.930 – 0.992]	0.984 [0.954 – 0.997]	13.7	38.2

AUROC, area under receiver operating characteristics curve.
Optimal cutoff values were determined using Youden index. Values in the square brackets represents 95% confidence intervals.
